# Practical Considerations for the Management of Cancer-Associated Venous Thromboembolism: A Guide for the General Oncology Practitioner

**DOI:** 10.3390/curroncol29090505

**Published:** 2022-09-08

**Authors:** Amye M. Harrigan, Josée Rioux, Sudeep Shivakumar

**Affiliations:** 1 Department of Medicine, Division of Hematology, Dalhousie University, Nova Scotia Health, Halifax, NS B3H 2Y9, Canada; 2 Department of Pharmacy, Nova Scotia Health, Victoria General Site, Halifax, NS B3H 2Y9, Canada

**Keywords:** cancer-associated venous thromboembolism, direct oral anticoagulant, low-molecular-weight heparin

## Abstract

Cancer-associated venous thromboembolism is a devastating complication of cancer and is associated with significant morbidity and mortality. The cornerstone of cancer-associated venous thromboembolism treatment is anticoagulation, and in recent years, there have been notable randomized clinical trials that have revealed insights into the efficacy and safety of direct oral anticoagulants and low-molecular-weight heparin in the treatment of cancer-associated thrombosis. Deciding on the ideal anticoagulation treatment plan for a patient with a cancer-associated thrombosis is a complex task that requires an understanding of clinical trial data, society guidelines, and, most importantly, consideration of many cancer-related, treatment-related, and patient-related factors. This article summarizes important factors to consider when deciding on anticoagulation therapy for a patient with cancer-associated thrombosis.

## 1. Introduction

Cancer-associated venous thromboembolism (CAT), which consists of deep vein thrombosis (DVT) and pulmonary embolism (PE), is a major cause of morbidity and mortality amongst patients with cancer [1]. The association between cancer and a hypercoagulable state has long been recognized, but the risk of thrombosis in patients with cancer is heterogeneous and dependent on several factors that can be categorized into patient-related, cancer-related, and treatment-related factors (Table 1). Advances in cancer treatments have led to improved patient outcomes over the years; however, the incidence of CAT continues to increase [2,3]. Compared to the general population, the 6-month VTE risk for patients with cancer is 12-fold higher, and it is estimated that CAT will affect 15-20% of patients with cancer throughout their lifetime [4,5]. Cancer-associated venous thromboembolism is also one of the most common causes of death in patients with cancer [6]. In a recent systematic review and meta-analysis of case fatality rates of recurrent VTE and major bleeding in patients with CAT, a case fatality rate of 14.8% for recurrent PE and a case fatality rate of 8.9% for major bleeding were reported in the initial 3-to-6-month anticoagulation treatment period [7]. As with any VTE event, the cornerstone of CAT treatment is anticoagulation therapy; however, choosing the most suitable anticoagulant treatment is more challenging in CAT, given the presence of numerous other factors related to cancer and its treatment. Over the last few years, there have been several important clinical trials examining the efficacy and safety of low-molecular-weight heparin (LMWH) and direct oral anticoagulants (DOACs). While these trials have revealed important information regarding the safety and efficacy of DOACs and LMWH in CAT treatment, choosing a suitable anticoagulation treatment plan for patients with CAT is complex and challenging as there is a multitude of factors to consider. The objective of this paper is to illustrate some of the important factors to consider when deciding on anticoagulation therapy for a patient with CAT.

## 2. Anticoagulation Options for the Management of CAT

The goal of anticoagulation treatment in CAT is two-fold: halt the extension of the clot and prevent the recurrence of VTE. Pharmacologic options for CAT treatment have historically consisted of unfractionated heparin (UFH), LMWH, and vitamin K antagonists (VKAs). For over a decade, LMWH has been the recommended anticoagulation agent for the management of CAT. The landmark CLOT trial and numerous subsequent trials have demonstrated that LMWH has greater efficacy in the prevention of thrombosis recurrence and a lower risk of bleeding compared to vitamin K antagonists such as warfarin [9,10,11,12,13,14]. LMWHs, such as enoxaparin, dalteparin, and tinzaparin, have been reported to have comparable effectiveness and safety profiles in the treatment of CAT [15]. While there is improved efficacy and safety with LMWH compared to oral VKAs, some significant disadvantages of LMWH include the burden of daily injections and the high cost of these anticoagulants [16,17].

The role of DOACs in the treatment of CAT has been the subject of many important randomized trials in the last few years. DOACs have advantages in this patient population as they have an oral route of administration and do not require extensive laboratory monitoring. The factor Xa inhibitors apixaban, edoxaban, and rivaroxaban, have each had at least one major randomized trial that evaluated their efficacy at preventing a recurrent VTE and safety profile with respect to bleeding complications compared to LMWH (dalteparin). In each of their respective trials, the studied DOAC has been found to have lower rates of recurrent VTE compared to LMWH (Table 2) [18,19,20,21,22,23]. However, there have been variable results as to whether DOACs are associated with a higher risk of major bleeding and clinically relevant non-major bleeding (CRNMB). In the HOKUSAI-VTE (edoxaban) and SELECT-D (rivaroxaban) trials, the studied DOAC had a higher risk of major bleeding and CRNMB compared to LMWH [18,19]. In comparison, the CARAVAGGIO trial (apixaban) was the first major trial in which the study DOAC (apixaban) was not associated with an increased risk of major bleeding episodes compared to LMWH [20]. In the smaller ADAM-VTE trial, apixaban was again found to have lower rates of major bleeding and similar rates of CRNMB compared to LMWH [21]. While an in-depth review of the differences between these randomized clinical trials is beyond the scope of this paper, it is important to recognize that each trial had its own unique inclusion criteria for patients, types of malignancy included, duration, and outcome measures that need to be considered when interpreting the results of the trial (Table 2).

A recent meta-analysis published by Frere et al. includes all the publicly available results from the randomized control trials comparing LMWH with DOACs for the treatment of CAT [24]. Six randomized control trials and a total of 3690 patients with acute CAT (i.e., 1850 randomized to the DOAC arms and 1840 randomized to the LMWH arms) were included in the meta-analysis. Results showed that the risk of recurrent VTE was significantly lower with DOACs compared to LMWH (RR 0.67; 95% CI, 0.52–0.85; *p* = 0.001; I^2^ = 0%) and the absolute risk reduction of recurrent VTE with DOACs was 2.7% (95% CI, −4 to −1.2) [24]. In terms of the risk of major bleeding, this was numerically higher with DOACs; however, the difference did not reach statistical significance (RR 1.17, 95% CI, 0.82–1.67; *p* = 0.39; I^2^ = 12%), and the absolute risk increase in major bleeding with DOACs was 0.6% (95% CI, from −0.7 to 2.5) [24]. Clinically relevant non-major bleeding occurred more frequently in patients receiving DOACs compared to those receiving LMWH (RR 1.66, 95% CI, 1.31–2.09; *p* < 0.0001; I^2^ = 0%) and the absolute risk increase in CRNMB with DOACs was 3.8% (95% CI, 1.8–6.2) [24]. Other interesting findings from this meta-analysis include that the proportion of patients discontinuing treatment was lower in those randomized to receive a DOAC compared to those randomized to receive LMWH and that overall, there was no difference in all-cause mortality rates between those who received LMWH or DOAC [24].

## 3. Important Considerations When Choosing an Anticoagulation Treatment for a Patient with CAT

With increasing evidence supporting the use of DOACs as an effective and safe treatment option for select patients with CAT, international and national societies have released clinical practice guidelines outlining recommendations to facilitate the evidence-based management of CAT [25,26,27,28,29]. While these guidelines are an excellent resource to help inform clinicians, it is imperative that clinicians recognize that determining the most suitable anticoagulation treatment plan for patients with CAT requires a thorough evaluation of patient-related, cancer-related, and treatment-related factors [25,26,27,28,29]. Of utmost importance, the decision around anticoagulation should be a shared decision between the patient and their physician. The following is an outline of some of the more important factors to consider when deciding on an anticoagulation treatment for a patient with CAT.

### 3.1. Burden and Type of Venous Thromboembolism Event

One of the first factors to consider when deciding on the type of anticoagulation for a CAT event is the location and burden of the VTE event. In patients who have severe or life-threatening presentations (e.g., iliofemoral DVT, sub-massive PE, or need for thrombolysis), treatment with LMWH is suggested as the initial anticoagulation treatment, and a DOAC can be considered when the patient’s clinical status has improved [25].

With the increased use of computed tomography (CT) and evolving imaging quality over the years, there has been an increase in the rates of VTE events detected incidentally on screening and surveillance imaging. Notably, incidental VTE events were included in all the major randomized control trials looking at LMWH vs. DOAC, and while the rate of recurrent VTE is lower in patients with incidental VTE compared to those with symptomatic VTE, the overall rate of recurrent events remains high [18,19,20,21,22,23,30,31]. As per national and international society guidelines, it is suggested that patients with incidental CAT receive the same initial and long-term anticoagulation as patients with symptomatic CAT [25,26,27,28,31].

Subsegmental pulmonary embolus (SSPE) is another entity that is more commonly detected, given the improvements in radiographic imaging. SSPE poses a particular treatment challenge as the clinical significance of SSPE, especially in a single SSPE in the absence of concomitant DVT, is not well characterized [32]. In a subgroup analysis of an international prospective cohort study involving patients with cancer and incidental PE, SSPEs and more proximal PEs had a similar 12-month recurrence rate of VTE (6.4% and 6.0%, respectively) [33]. Recommendations for the management of multiple SSPEs in patients with cancer are consistent across different societal guidelines [26,27,34]. It is suggested that patients with multiple SSPEs receive treatment with therapeutic anticoagulation similar to that of a proximal incidental or symptomatic PE [26,27,34]. If an isolated SSPE is found in a patient with cancer, an ultrasound of both lower extremities should be performed to detect the presence of a concomitant DVT, and the decision to start therapeutic anticoagulation should be made on a case-by-case basis taking into consideration the presence or absence of concomitant DVT as well as usual bleeding risk factors [26,27,34].

Another type of VTE event encountered in patients with cancer is the unusual site of thrombosis (such as thrombosis in major abdominal or pelvic veins or cerebral venous thrombosis) [35]. There are limited data to guide the efficacy, safety, and optimal duration of catheter-related and unusual site VTE in cancer patients, as these events were excluded from most of the randomized trials. The one exception was in the ADAM-VTE trial, which enrolled individuals with unusual site thrombosis (Table 2) [21]. The only clinical practice guideline to publish guidance on the management of unusual site thrombosis is from the National Comprehensive Cancer Network (NCCN), which provides guidance around the treatment of splanchnic vein thrombosis (SVT) [36]. The NCCN guidelines have made a weak recommendation that patients with cancer and an acute (i.e., signs/symptoms within 8 weeks or less) SVT should receive anticoagulation if there are no contraindications to anticoagulation [36]. This recommendation for the treatment of acute (e.g., 8 weeks or less) symptomatic splanchnic vein thrombosis is also consistent with recommendations made by other societal guidelines and expert consensus for the treatment of SVT in non-cancer patients [35]. As for CAT at other unusual sites, clinical practice guidelines state there is inadequate evidence to make recommendations for the treatment of these unusual site thrombosis and advise that factors such as diagnostic certainty, chronicity, the extent of thrombosis, and associated symptoms should be considered when deciding to start anticoagulation treatment [26,27]. In the non-CAT unusual site thrombosis literature, treatment of cerebral venous sinus thrombosis (CVST) with unfractionated intravenous heparin or LMWH in the acute phase is recommended by societal thrombosis and neurology guidelines, and therefore, this may represent another unusual thrombosis site that warrants treatment in patients with CAT [35].

Central venous catheters are frequently placed for the administration of chemotherapy, blood products, parenteral nutrition, and other therapies in patients with cancer. It is estimated that the rates of catheter-related thrombosis in patients with cancer are 14–18% and 5% for asymptomatic and symptomatic events, respectively [37]. Recognized catheter-related factors that may increase the occurrence of catheter-related VTE include factors related to the location of the catheter, presence of catheter-related fibrin sheath, and type of catheter placed [37,38,39]. An increased risk of catheter-related thrombosis has been reported with left-sided catheters, placement of the catheter tip above the junction between the superior vena cava and the right atrium, improper catheter tip placement as well as with peripherally implanted central venous catheters (PICCs) and catheter with multiple lumens, as well as patients with a high BMI and non-breast cancers [37,38,39,40,41]. For the treatment of catheter-related thrombosis in patients with cancer, society guidelines suggest that the choice of agent, DOAC or LMWH, should be individualized similarly to other CAT events and that duration should be at least 3 months or for as long as the catheter remains in place [25,26,27,28,29,38,42]. The catheter does not need to be removed and can continue to be used if it is functioning properly and when there is no sign of infection [25,26,27,28,29,38,42].

Recurrent VTE can be encountered in the context of CAT and may be due to factors such as cancer progression, interruptions in anticoagulation therapy, or new risk factors for thrombosis such as insertion of a central venous catheter or new anticancer treatment [43]. Patients with recurrent VTE despite standard doses of anticoagulant therapy should be assessed for treatment compliance and heparin-induced thrombocytopenia (HIT), and repeat radiographic imaging should be performed to confirm the new thrombosis by comparing it to previous imaging, as well as assess for any evidence of mechanical compression resulting from malignancy or progression of malignancy [43]. There are limited studies on the management of recurrent VTE in a patient with CAT, but strategies described in the clinical guidelines include switching to an LMWH if on a DOAC or another oral anticoagulant was initially used or, if already on LMWH, increasing the dose of LMWH by 20 to 25% (i.e., supratherapeutic dose) [26,27].

### 3.2. Cancer Type

The data from the randomized control trials comparing DOACs to LMWH in the treatment of CAT illustrate the need to consider the type of cancer when assessing bleeding risk in patients who require anticoagulation. In the HOKUSAI-VTE and SELECT-D trials, the study DOAC was associated with an increased risk of major bleeding compared to dalteparin [18,19]. Looking closer at the major bleeding episodes, the majority of these were gastrointestinal (GI) in nature and occurred predominantly in patients with GI malignancies, particularly those with upper GI tract cancers [18,19]. Conversely, the CARAVAGGIO trial did not show an increased risk of GI bleeding events with apixaban compared with dalteparin (LMWH) [20]. The increased risk of GI bleeding in patients with luminal GI cancer is postulated to be due to a combination of factors, including the anticoagulant effect of the DOAC on the GI tract after ingestion, the luminal tumor, and the presence of other non-neoplastic GI lesions such as esophagitis or peptic/duodenal ulcers or arteriovenous vascular malformations [44]. Taking these data into consideration, DOACs should be used cautiously in patients with GI cancers, especially if the luminal tumor has not been resected or if a patient’s anticancer therapy places them at risk of GI perforation and/or hemorrhage [25,26,27,28].

Genitourinary (GU) cancers involving the kidney, urothelial tract, or bladder are another type of cancer where LMWH is the preferred anticoagulation agent for the treatment of CAT. In the HOKUSAI VTE study subgroup analyses for major bleeding, GU cancers were also a group that was found to have a higher risk of major bleeding when receiving edoxaban compared to dalteparin [18]. Furthermore, meta-analyses have reported that there are significantly increased rates of major GU bleeding and increased rates of GU CRNMB in patients receiving DOACs compared to LMWH [45,46]. Given the findings of increased risk of GU major bleeding and CRNMB and that patients with active, unresected lesions in the GU tract, recent GU tumor surgery, or those with instrumentation within the GU tract (e.g., urinary stents, nephrostomy tubes) would be those at highest risk of GU bleeding, it is suggested that these patients receive LMWH instead of a DOAC [25,26,27,28,29].

Intracranial lesions from a primary CNS tumor or metastases also warrant particular attention when choosing anticoagulation for CAT. While intracranial tumors are associated with an increased risk of VTE, there is also the risk of intracranial hemorrhage (ICH) [47,48,49]. In the major DOAC trials for the treatment of CAT, patients with intracranial malignancy (primary or metastatic) were either excluded from the trials or only represented a small proportion of the study population [18,19,20,21,22,23]. In the studies that did include patients with primary or metastatic brain tumors, subgroup analyses did not show any significant differences between the rates of major bleeding or recurrent VTE [18,19,21,23]. Retrospective studies looking at anticoagulation in patients with malignant intracranial lesions have provided further insight into the use of LMWH and DOACs for the treatment of CAT. In a meta-analysis looking at the rates of ICH in patients with brain tumors and CAT receiving LMWH compared to no anticoagulation, patients with metastatic brain tumors on LMWH did not have an increased risk of ICH compared to those who did not receive anticoagulation [48]. There was, however, an increased risk of ICH in patients with high-grade glioma [48]. A subsequent matched cohort study of patients with high-grade glioma did confirm that patients with high-grade glioma had a 3.37-fold increase in the risk of major ICH when receiving enoxaparin (LMWH) compared to those who did not receive anticoagulation, but the latter group had an 11-fold increase in the risk of recurrent VTE [49]. As for the use of DOACs compared to LMWH in this CNS malignancy population, the results from small, retrospective cohort studies are mixed, with one study reporting no difference in ICH between DOACs and LMWH treatments while the other reported that patients receiving DOACs had a lower rate of ICH compared to those receiving LMWH [47,49]. Given the limitations of the available data, it can be challenging to choose between LMWH and DOACs for the treatment of CAT in patients with intracranial malignancies but important to consider that although DOACs appear to be a least as safe as LMWH in patients with intracranial malignancy, it is suggested that patients with certain intracranial lesions with a high bleeding risk such as gliomas or metastases from renal cell carcinoma or melanoma receive LMWH given the shorter half-life compared to DOACs [25,27,34].

### 3.3. Pharmacologic and Organ Function Considerations

Understanding the pharmacologic properties of DOACs and LMWH and their potential for interactions with anticancer therapies, supportive medications, and a patient’s other medications is another important consideration when selecting an anticoagulation treatment. Despite its inconvenient mode of administration, LMWH has the benefit of having fewer drug–drug interactions compared to DOACs and does not require absorption through the GI tract [44]. In comparison, DOACs are a substrate for P-glycoprotein (P-gp), a transporter that mediates drug absorption and excretion, and rivaroxaban and apixaban are also strongly dependent on the CYP3A4 system in the liver for metabolism [44,50]. There are published comprehensive reviews outlining the theoretical increased risk between DOACs and various anticancer agents; however, there are limited data in the real-world setting outlining the outcomes of these drug–drug interactions [51,52,53,54]. Clinicians should be aware of the potential for drug–drug interactions that could lead to supra- or sub-therapeutic levels of DOAC, and, when available, a pharmacist-led drug–drug interaction evaluation should be completed prior to starting a patient on anticoagulation or when there is a change in the patient’s cancer treatment. Table 3 lists some clinically important drug interactions with DOACs.

Factors that impact a patient’s oral intake and absorption are also important to consider prior to starting a patient on a DOAC. The most frequently encountered issue that affects oral intake in patients with cancer is nausea and/or vomiting, especially when receiving highly emetogenic anticancer treatment [55]. In particular, the bioavailability and part of the absorption of rivaroxaban are dependent on the acidic environment of the stomach and, therefore, must be taken with food [56]. Altered GI tract anatomy should also be considered as surgical resection of the stomach, small bowel, or ascending colon is hypothesized to lead to reduced DOAC absorption [25,57]. However, there are limited real-world data on the outcomes of patients with altered GI tract anatomy treated for CAT with a DOAC [25,57].

A cancer patient’s physiological status and its impact on the distribution of a drug, especially for DOACs, is another important consideration when choosing an anticoagulant. Poor appetite, cachexia, and emetogenic anticancer treatments often result in sarcopenia, hypoalbuminemia, and reduced lean body mass, all of which contribute to a reduced volume of distribution of DOACs, higher plasma concentrations, and consequently increased risk of bleeding [58,59]. Reduced lean body mass is also a normal physiologic change of aging, which places underweight, older patients with CAT at a particularly increased risk of having higher plasma levels of DOACs [58,59]. A patient’s low body weight (i.e., 60 kg or less) should be recognized as a risk factor when assessing a patient’s bleeding risk prior to starting anticoagulation therapy [59]. One should also remember to account for low body weight when calculating a patient’s renal function, as low body weight may lead to an overestimate of a patient’s renal function (CrCl) and can also lead to increased bleeding risk while receiving anticoagulation therapy (see paragraph on renal function below for further details) [59]. If a DOAC is chosen for a patient with low body weight, apixaban or edoxaban should be considered, as both DOACs have recommended dose reductions based on low body weight (weight of 60 kg or less) [59].

The renal and hepatic functions also need to be considered when choosing an anticoagulation agent for the treatment of CAT. Both LMWH and DOACs rely on the kidneys and/or liver for elimination [60]. In all the CAT DOAC vs. LMWH randomized clinical trials, patients with a creatinine clearance (CrCl) below 30 mL/min or with significant liver dysfunction (i.e., Child–Pugh class C) were excluded, and thus, the conclusions from these trials are not applicable to these patients [18,19,20,21,22,23]. From a renal function perspective, there are certain agents that are approved for cautious use in patients with CrCl < 30 mL/min. Both apixaban and rivaroxaban have been approved for use with caution in patients with CrCl of 15–29 mL/min, with the caveat that there is a potentially higher risk of bleeding [25]. Similarly, LMWH enoxaparin and tinzaparin are approved for use in individuals with CrCl of <15 mL/min and dialysis [25]. In liver disease, DOACs should not be used in individuals with Child–Pugh C, and, for individuals with Child–Pugh B liver disease, edoxaban is perhaps the preferred DOAC as patients with Child–Pugh A or B have been shown to have similar pharmacokinetics and pharmacodynamics to healthy controls [25]. Overall, society guidelines suggest that a chosen anticoagulant’s product monograph be reviewed to determine if the chosen anticoagulant is safe to use given the patient’s renal or liver function and if any dose adjustments are needed [25,26,27,28].

Thrombocytopenia due to underlying malignancy or related to anticancer treatment is another challenge faced in the management of anticoagulation for CAT. Patients with a platelet count < 50 × 10^9^ g/L are at risk of bleeding, especially if on anticoagulation, but they are also at increased risk of recurrent VTE [61]. A platelet count < 50 × 10^9^ g/L was an exclusion criterion in all the major CAT trials, and there are few studies that evaluate the optimal anticoagulation strategy in this patient population. In a guidance document released by the International Society of Thrombosis and Hemostasis (ISTH), LMWH is the preferred anticoagulant for the treatment of CAT in the context of thrombocytopenia [61]. Furthermore, the acuity and burden of thrombosis should be considered when choosing an anticoagulation strategy in the setting of thrombocytopenia. In patients with high-risk features such as symptomatic segmental or more proximal PE, proximal deep vein thrombosis (DVT), or a history of recurrent/progressive thrombosis, therapeutic doses of anticoagulation with platelet transfusion support to maintain platelet counts above 40–50 × 10^9^ g/L should be considered in the acute thrombosis period (i.e., the first 30 days from diagnosis) [61]. For patients with lower-risk events, a dose-modification strategy using 50% or prophylactic-dose LMWH for patients with platelet counts of 25–50 × 10^9^ g/L and holding the anticoagulation if the platelet count falls below 25 × 10^9^ g/L may be considered [61]. After 30 days of treatment, the risk of VTE recurrence is decreased, and lower-dose or modified-dose anticoagulation can be considered to reduce the risk of bleeding and avoid a transfusion burden [61].

### 3.4. Patient Characteristics, Preferences, and Drug Coverage

Patient involvement in the decision-making process is of utmost importance when choosing an anticoagulation treatment. Qualitative studies examining the experiences of patients with CAT have reported that a diagnosis of CAT adds an additional burden to an already challenging and often frightening journey through cancer [62,63]. Each patient will have their own unique set of values and preferences, which can help inform the decision between a DOAC or LMWH for the treatment of CAT. Qualitative studies conducted prior to DOACs being widely used in the treatment of CAT have highlighted that the daily injections required with LMWH therapy were an “acceptable” intervention [63,64]. These studies reported that patients with CAT placed a higher value on an anticoagulant that had minimal interference with their anticancer treatment, had a low thrombosis recurrence rate, and had a low risk of major bleeding, while an oral mode of administration was only of moderate importance [63,64]. Considering the more recent data demonstrating the efficacy and safety of DOACs in the treatment of CAT, further qualitative studies have re-examined patient preference for the treatment of CAT. In these recent studies, patients reported increased treatment satisfaction with DOACs compared to LMWH [65,66]. Similar findings were also reported in the secondary outcomes of the ADAM-VTE trial and a sub-analysis of the SELECT-D trial [21,67].

Prior to providing a patient with a prescription for an anticoagulation agent, clinicians should enquire about the type of drug coverage a patient has, as well as understand the potential costs that may be associated with the anticoagulation agent of choice. Most Canadians have access to some form of drug coverage through either a private drug plan and/or a provincial or territorial government plan (Table 4 and Table 5). There are variations in the eligibility criteria for the coverage depending on the province or territory; however, anticoagulation agents (LMWH or DOAC) are funded for the guideline-recommended 6-month treatment duration [25,26,27,28,29]. Coverage for DOAC is up to 6 months duration, with Quebec offering up to 12 months in some situations (Table 4 and Table 5). As for LMWH, many provinces have them listed as regular benefits (with no restrictions), while other provinces have duration limits of 3 months, up to a year (Table 4 and Table 5). It is important to keep in mind that while these medications are listed on the provincial formulary, depending on the plan, the co-pay or deductible may still pose a barrier to the patient acquiring the drug.

## 4. Conclusions

Cancer-associated thrombosis is a common and devastating complication of cancer. The management of CAT is a complex task that requires careful evaluation of the clinical trial evidence on the efficacy and safety of anticoagulation agents, in addition to considering many other patient-related and cancer-related factors. Finally, it is also important to remember that the most suitable anticoagulation plan may change as a patient continues along their journey through cancer.

## Figures and Tables

**Table 1:** Clinical risk factors for cancer-associated venous thromboembolism (adapted from Gervaso et al.) [8].

Patient-Related Factors	Cancer-Related Factors	Treatment-Related Factors
Older age	Initial diagnosis	Major surgery
Ethnicity	Primary cancer - Brain - Kidney - Gastrointestinal - Lung - Gynecologic - Hematological (esp. lymphoma and myeloma)	Hospitalization
Female		Cancer therapy - Radiotherapy - Chemotherapy - Hormonal therapy - Immunomodulatory agents (e.g., thalidomide, lenalidomide) - Antiangiogenic agents (e.g., bevacizumab) - Immune check point inhibitors
Comorbidities - Obesity - Renal disease, - Infection, - Inherited thrombophilia		
Prior history of VTE	Advanced cancer stage	Central venous catheters
Poor performance status	Cancer histology (e.g., adenocarcinoma)	Transfusions

**Table 2:** Randomized controlled trials for the acute treatment of cancer-associated thrombosis.

Study Name	Study Design; Patients Enrolled (N); Duration (Months)	Study Population	Definition of Cancer	Three Most Common Cancer Types	Notable Excluded Malignancies	LMWH DOAC	DOAC	LMWH
HOKUSAI-VTE CANCER [18]	Open-label, randomized, non-inferiority trial N = 1050 12 mo	Adults with active cancer and acute symptomatic or incidentally discovered DVT of the lower leg or PE Incidental PE: segmental or involving more proximal pulmonary arteries	Active cancer: - New diagnosis within the previous 6 months - Recurrent, regionally advanced, or metastatic cancer - Cancer treatment within 6 months - Hematologic cancer not in complete remission	Colorectal Lung Genitourinary	NA	Dalteparin Edoxaban LMWH given for at least 5 days at start of treatment	VTE recurrence 7.9%	VTE recurrence 11.3%
							HR: 0.71 (95% CI, 0.48–1.06) *p*-value 0.09	
							MB 6.9%	MB 4.0%
							HR: 1.77 (95% CI, 1.03–3.04) *p*-value 0.04	
							CRNMB 14.6%	CRNMB 11.1%
SELECT-D [19]	Open-label, randomized pilot trial N = 203 6 months	Active cancer presenting with a primary objectively confirmed VTE symptomatic lower-extremity proximal DVT, symptomatic PE, or incidental PE	Active cancer: - New diagnosis of cancer (solid or hematologic) in the last 6 months - Cancer treatment in the last 6 months - Recurrent or metastatic cancer, or cancer not in complete remission (heme malignancy)	Colorectal Lung Breast	Protocol amendment during the study period to exclude patients with esophageal or gastroesophageal because of high rates of GI bleeding	Dalteparin Rivaroxaban	VTE recurrence 4.0%	VTE recurrence 11.0%
							HR, 0.43 (95% CI, 0.19 to 0.99)	
							MB 6.0%	MB 4.0%
							HR: 1.83 (95% CI, 0.68 to 4.96)	
							CRNMB 13%	CRNMB 4%
ADAM VTE [21]	Open-label, randomized, superiority trial N = 283 6 months	Acute thrombosis including lower extremity or upper extremity DVT, PE, splanchnic, or cerebral vein thrombosis confirmed by appropriate cross-section imaging	Active cancer: - Cancer on cross-sectional or positron emission tomography imaging - Metastatic disease - Cancer-related surgery, chemotherapy, or radiation therapy in the last 6 months	Colorectal Lung Pancreatic	No specific cancer types (inc. brain metastasis) were excluded	Dalteparin Apixaban	VTE recurrence 0.7%	VTE recurrence 6.3%
							HR: 0.099 (95% CI 0.013–0.78) *p*-value 0.0281	
							MB 0%	MB1.4%
							HR: not estimable because of 0 bleeding event in apixaban group *p*-value 0.138	
							CRNMB 6.2%	CRNMB 4.2%
CARVAGGIO [20]	Open-label, non-inferiority, randomized trial with blinded central outcome adjudication N = 1700 6 months	Adults with cancer and newly diagnosed symptomatic or incidental proximal lower-limb DVT or PE	Active cancer: - Cancer diagnosed within the past 6 months - Cancer treatment in the last 6 months - Recurrent locally advanced or metastatic cancer	Colorectal Lung Breast	Primary brain tumors Intracerebral metastasis Acute myeloid Llukemia	Dalteparin Apixaban	VTE recurrence 5.6%	VTE recurrence 7.9%
							HR: 0.63 (95% CI 0.37 to 1.07) *p* < 0.001 for noninferiority; *p*-value 0.09 for superiority)	
							MB 3.8%	MB 4.0%
							HR: 0.8 (95% CI, 0.40 to 1.69) *p*-value 0.6	
							CRNMB 9.0%	CRNMB 9.0%
CASTA-DIVA [23]	Open-label, non-inferiority, randomized trial N = 158 3 months	Adult cancer patients with newly diagnosed symptomatic or incidental proximal lower-limb DVT, symptomatic or incidental iliac or inferior vena cava thrombosis or PE, or both and high risk of recurrent VTE despite anticoagulation as estimated by a modified Ottawa score of ≥1	Solid cancer, high-grade lymphoma or thalidomide, or lenalidomide-treated myeloma	Colorectal Lung Brest	NA	Dalteparin Rivaroxaban	VTE recurrence 6.4%	VTE recurrence 10.1%
							HR: 0.75 (95% CI, 0.21–2.66) *p*-value 0.13 for non-inferiority	
							MB 1.4%	MB 3.7%
							HR: 0.36 (95% CI, 0.04–3.43)	
							CRNMB 12.2%	CRNMB 9.8%
CANVAS [22] * presented as abstract at ASCO	Pragmatic trial, unblinded hybrid comparative effectiveness non-inferiority trial Randomized and preference cohorts N = 671 randomized cohort N = 140 preference cohort 6 months	Adults with any invasive solid tumor, lymphoma, multiple myeloma, or CLL and a diagnosis of symptomatic or radiographically detected VTE within 30 days	Solid tumor, lymphoma, multiple myeloma, or CLL	NR	NA	Any LMWH Any DOAC	VTE recurrence 6.4%	VTE recurrence 7.8%
							HR: NR	
							MB 5.4%	MB 4.4%
							HR: NR	
							CRNMB: NR	CRNMB: NR

* CLL=chronic lymphocytic leukemia, CRNMB = clinically relevant non-major bleeding, DVT= deep vein thrombosis, HR = hazard ratio, MB = major bleeding, NA = not applicable, NR= not reported, PE = pulmonary embolism, SSPE = subsegmental pulmonary embolism, VTE = venous thromboembolism.

**Table 3:** Clinically significant drug–drug interactions with DOACs (adapted from Carrier et al. 2021) [25].

Outcome	Drug
**Increase bleeding risk**	**Antiarrhythmic/antihypertensive agents**: amiodarone, diltiazem, quinidine, verapamil
	**Antimicrobials/antifungals**: clarithromycin, fluconazole, miconazole
	**Immunosuppressants**: cyclosporine
	**Anti-diarrhea agent**: loperamide
	**Tyrosine kinase inhibitors**: acalabrutinib, ibrutinib
**Decrease antithrombotic efficacy**	**Anticonvulsants**: carbamazepine, oxcarbazepine, phenobarbital
	**Antimicrobial/antiviral**: efavirenz, nevirapine, rifampin
	**Monoclonal antibody**: Tocilizumab

**Table 4:** Coverage of DOACs for venous thromboembolism under provincial/territorial government drug plans.

Anticoagulant	Province/Territory												
	BC	AB	SK	MB	ON	QC	NB	NS	PEI	NL	NWT	YT	NU
Apixaban	✓	✓	✓	✓	✓	✓	✓	✓	✓	✓	✓	✓	✓
Rivaroxaban	✓	✓	✓	✓	✓	✓	✓	✓	✓	✓	✓	X	✓
Edoxaban	X	✓	✓	✓	✓	✓	✓	✓	✓	✓	✓	✓	✓

Venous thromboembolism (VTE) = deep vein thrombosis (DVT) and pulmonary embolism (PE). ✓ Covered under Provincial/Territorial Government Drug Plan—for the treatment of VTE for up to six months (special authorization form typically required). **X** Not covered under Provincial/Territorial Government Drug Plan for VTE. 1. Apixaban only in Quebec—for *idiopathic* VTE, can apply for long-term coverage for prevention of recurring VTE in persons who were treated with anticoagulation therapy during a period of at least six months for an acute idiopathic VTE (approval must be renewed every 12 months). 2. Edoxaban only in Quebec—for the treatment of VTE for up to 12 months.

**Table 5:** Coverage of LMWH for VTE under provincial/territorial government drug plans.

Province/Territory Criteria for LMWH Coverage for VTE												
BC	AB	SK	MB	ON	QC	NB	NS	PEI	NL	NWT	YT	NU
6 mo for cancer patients	Regular benefit	Long-term coverage if CI to warfarin *	✓	1 yr if CI to warfarin *	Regular benefit	6 mo for cancer patient	Regular benefit	6 mo for cancer patient	3 mo for acute Tx in cancer patient	Regular benefit	Regular benefit	Regular benefit

LMWH = lower molecular weight heparins (dalteparin, enoxaparin, tinzaparin), mo = month, CI = Contraindication, yr = year, Tx = treatment. * Patients on active cancer treatment likely to be considered as a relative contraindication for warfarin. ✓ LWMH on formulary—internal coverage criteria, special authorization required.
